# Detecting early‐warning signals of influenza outbreak based on dynamic network marker

**DOI:** 10.1111/jcmm.13943

**Published:** 2018-10-19

**Authors:** Pei Chen, Ely Chen, Luonan Chen, Xianghong Jasmine Zhou, Rui Liu

**Affiliations:** ^1^ School of Mathematics South China University of technology Guangzhou China; ^2^ Department of Pathology and Laboratory Medicine David Geffen School of Medicine University of California at Los Angeles Los Angeles California; ^3^ Canadian Academy Kobe Japan; ^4^ Key Laboratory of Systems Biology CAS Center for Excellence in Molecular Cell Science Institute of Biochemistry and Cell Biology Shanghai China; ^5^ CAS Center for Excellence in Animal Evolution and Genetics Chinese Academy of Sciences Kunming China

**Keywords:** city network, critical transition, dynamic network biomarker (DNB), dynamic network marker (DNM), early‐warning signal, influenza outbreak

## Abstract

The seasonal outbreaks of influenza infection cause globally respiratory illness, or even death in all age groups. Given early‐warning signals preceding the influenza outbreak, timely intervention such as vaccination and isolation management effectively decrease the morbidity. However, it is usually a difficult task to achieve the real‐time prediction of influenza outbreak due to its complexity intertwining both biological systems and social systems. By exploring rich dynamical and high‐dimensional information, our dynamic network marker/biomarker (DNM/DNB) method opens a new way to identify the tipping point prior to the catastrophic transition into an influenza pandemics. In order to detect the early‐warning signals before the influenza outbreak by applying DNM method, the historical information of clinic hospitalization caused by influenza infection between years 2009 and 2016 were extracted and assembled from public records of Tokyo and Hokkaido, Japan. The early‐warning signal, with an average of 4‐week window lead prior to each seasonal outbreak of influenza, was provided by DNM‐based on the hospitalization records, providing an opportunity to apply proactive strategies to prevent or delay the onset of influenza outbreak. Moreover, the study on the dynamical changes of hospitalization in local district networks unveils the influenza transmission dynamics or landscape in network level.

## 1 INTRODUCTION

Despite current approaches to prevention and control, seasonal influenza remains a significant cause of morbidity and mortality worldwide.^1^ Being infected by influenza virus, people especially elderly and children are at a high‐risk for further deterioration including circulatory diseases, severe respiratory illness, and other life threatening complications.^2^, ^3^ Influenza pandemic also causes considerable economic burden including direct medical costs and indirect loss such as substantial workplace absenteeism. The estimated average direct medical costs of influenza in the United States reaches $10.4 billion each year,^4^ and the actual annual cost would be more.

Early detection and recognition of upcoming influenza outbreak, and timely public health prevention including vaccination schedule and control strategy, are critical in reducing the pandemic magnitude and distribution.^5^, ^6^ However, it is usually a challenging task to achieve the real‐time prediction of influenza outbreak due to its complex dynamics involving both biological systems and social systems. In addition, surveillance capacity for such detection can be costly, and many countries lack the public health infrastructure to identify outbreaks at their earliest stages. Furthermore, there may be economic incentives for countries to not fully disclose the nature and extent of an outbreak.^7^, ^8^ Therefore, a new computational method is required to predict the outbreak of epidemic diseases only based on available data, thus simplifying information gathering and monitoring processes.

**Figure 1:**
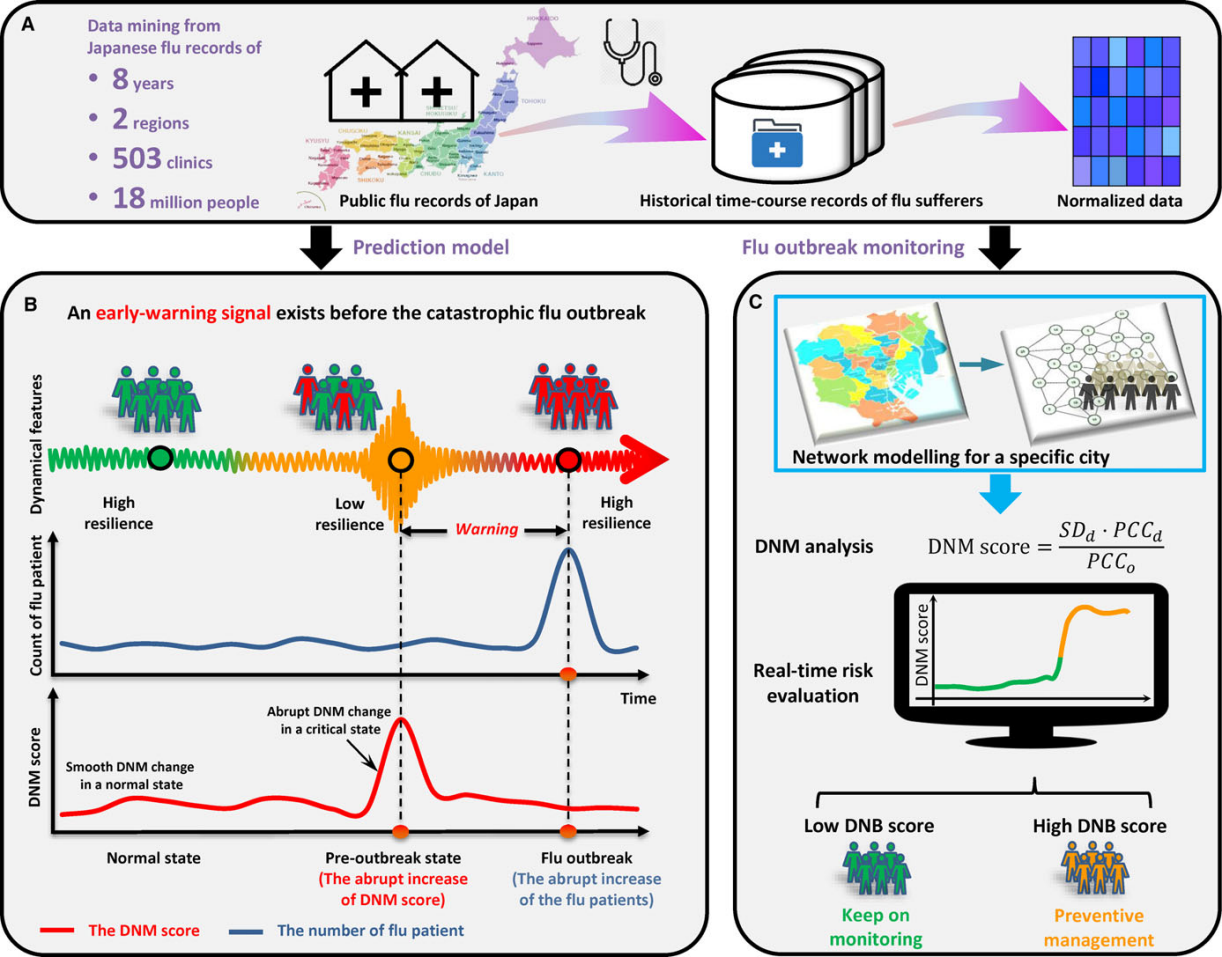
Schematic illustration to detect early‐warning signals of influenza outbreak based on the DNM method. A, The historical information of clinic hospitalization caused by influenza infection between 1 January 2009 and 31 December 2016 was extracted and assembled from public records of Tokyo and Hokkaido, Japan. B, According to the DNM theory, the process of a time‐dependent non‐linear system is divided into three states, including a normal state, a pre‐outbreak state and an after‐outbreak state. The abrupt increase in the DNM score indicates the pre‐outbreak state, ie, the tipping point just before the upcoming catastrophic influenza outbreak that results in a boost of clinic‐visiting patients. C, Based on the historical and current clinic records, and regional geographic characteristics of a city, the DNM score is able to provide the early‐warning signals of the upcoming influenza outbreak as a real‐time indicator monitoring

The dynamic network marker/biomarker (DNM/DNB) is our recently proposed method. It is a generalized methodology to identify the tipping point or pre‐transition state which is a critical state before the catastrophic event,^9^, ^10^ by mining the dynamical information from both horizontal high‐dimensional data and longitudinal historical records. Regarding the influenza outbreak as a tipping point at which the system undergoes a critical transition, then there is a common understanding that the dynamical process of the system can generally be expressed by three states (Figure 1B), that is, a normal state with high resilience, a pre‐outbreak state (the critical state) with low resilience, and an after‐outbreak state with possible high resilience. The normal state is a steady stage, during which there are no many clinic visiting patients. The pre‐outbreak state is defined as the limit of the normal state immediately before the tipping point. In this pre‐outbreak stage, the process is usually reversible to the normal state if appropriately treated, implying the criticality of the pre‐outbreak state. Unlike the traditional detection of the after‐outbreak state, the DNM enables the identification of the pre‐outbreak state or critical state that generally has no clear abnormalities but with future trending of deterioration or critical transition. This method has recently been successfully applied to a variety of biological progresses to detect the early‐warning signals to an irreversible catastrophic stage, such as the cell differentiation process,^11^ the process of cell fate decision,^12^ the critical transition in the immune checkpoint blockade‐responsive tumour,^13^ the multi‐stage deteriorations of T2D,^14^ acute lung injury,^15^ HCV induced liver cancer,^16^ cancer metastasis,^17^ and many others.^18^, ^19^, ^20^, ^21^ In this study, DNM method was employed to explore the dynamical information based on a combination of city network and the high‐dimensional clinic hospitalization records, which are from over 278 clinics distributed in 23 wards in Tokyo, Japan, and 225 clinics distributed in 30 districts in Hokkaido, Japan. The results show that the DNM method successfully identified the critical state just before the outbreak of influenza as a real‐time surveillance system. Such a system may enable a rapid response for the preventive care or the implementation of interventions to a health epidemic. In addition, this work unveils the influenza transmission dynamics or landscape in a local district network level, based on the measured data. The advantage and effectiveness of the DNM‐based system is also demonstrated by the comparison between DNM and other surveillance systems of flu pandemic.

## 2 MATERIALS AND METHODS

### 2.1 Dynamical network marker or dynamic network biomarker

Influenza viruses circulate around the world every year, causing financial losses, suffering, and death. The dynamical process of flu outbreak can be modeled by three states or stages (Figure 1) similar to disease progression^9^: the before‐outbreak state, which is a stable state with high resilience or high robustness to perturbations; the pre‐outbreak/critical state, which is the tipping point just before the catastrophic shift into the outbreak state and is thus characterized by low resilience or low robustness due to its critical dynamics, but is still reversible to the before‐outbreak state with appropriate control management; and the outbreak state, which is another stable state with high resilience or high robustness. Clearly, it is of great importance to identify the pre‐outbreak state, which holds the key to apply effective control management to prevent the catastrophic flu outbreak.

However, different from the outbreak state in which there are obvious signs including huge amount of outpatient visits, it is a difficult task to identify the pre‐outbreak state because there are generally no significant signs or differences between the before‐outbreak state and the pre‐outbreak state. On the other hand, the dynamic network marker/biomarker (DNM/DNB) method was developed to quantitatively identify the tipping point or the critical state during the dynamic evolution of a complex system based on the observed data. Theoretically, when a complex system is near the critical point, there exists a dominant group (a dominant group of variables or members) defined as the DNM features, which satisfy the following three necessary conditions based on the observed data^9^:

- The correlation (PCC_in_) between any pair of members in the DNM group rapidly increases;
- The correlation (PCC_out_) between one member of the DNM group and any other non‐DNM member rapidly decreases;
- The standard deviation (SD_in_) or coefficient of variation for any member in the DNM group drastically increases.

In other words, the above conditions can be approximately stated as: the appearance of a strongly fluctuating and highly correlated group of features implies the imminent transition into the flu outbreak. Then, these three conditions are adopted to quantify the tipping point as the early‐warning signals of diseases, and further, the identified dominant group of features consists of DNM members. The 2‐fold change threshold is usually applied to recognize the significant changes in DNM score and obtain the warning signal. The DNM theory has been applied to a number of analyses of disease progression and biological processes to predict the critical states as well as their driven factors.^9^, ^10^, ^11^, ^12^, ^13^, ^14^, ^15^, ^16^, ^17^, ^18^, ^19^, ^20^, ^22^ In this work, by considering the flu outbreak process as a non‐linear dynamics process, we further applied the DNM method to detect the tipping point or the early‐warning signal of flu outbreak. To quantify the critical state, the following criterion *I*_*DNM*_ was used as the signal of the critical point by combining the above three statistical conditions:

$$I_{\mathrm{DNM}}=\frac{PCC_{\mathrm{in}}}{PCC_{\mathrm{out}}}SD_{\mathrm{in}}.$$

Thus, from the observed data of a sample, whenever there is a group of features appearing with a high *I*_*DNM*_ score, this group of features is the DNM group and the state of this sample is considered to be near the tipping point. Therefore, from the hospitalization records of each sample, we can identify the DNM members and further quantify whether or not this sample is near the critical state using the *I*_*DNM*_ score.

To further reliably identify the critical state of flu outbreak, we developed a new method called the landscape DNM, which explores both the local and global records as well as the network structure, and the detailed algorithm is provided below.

### 2.2 Landscape DNM score

**Figure 2:**
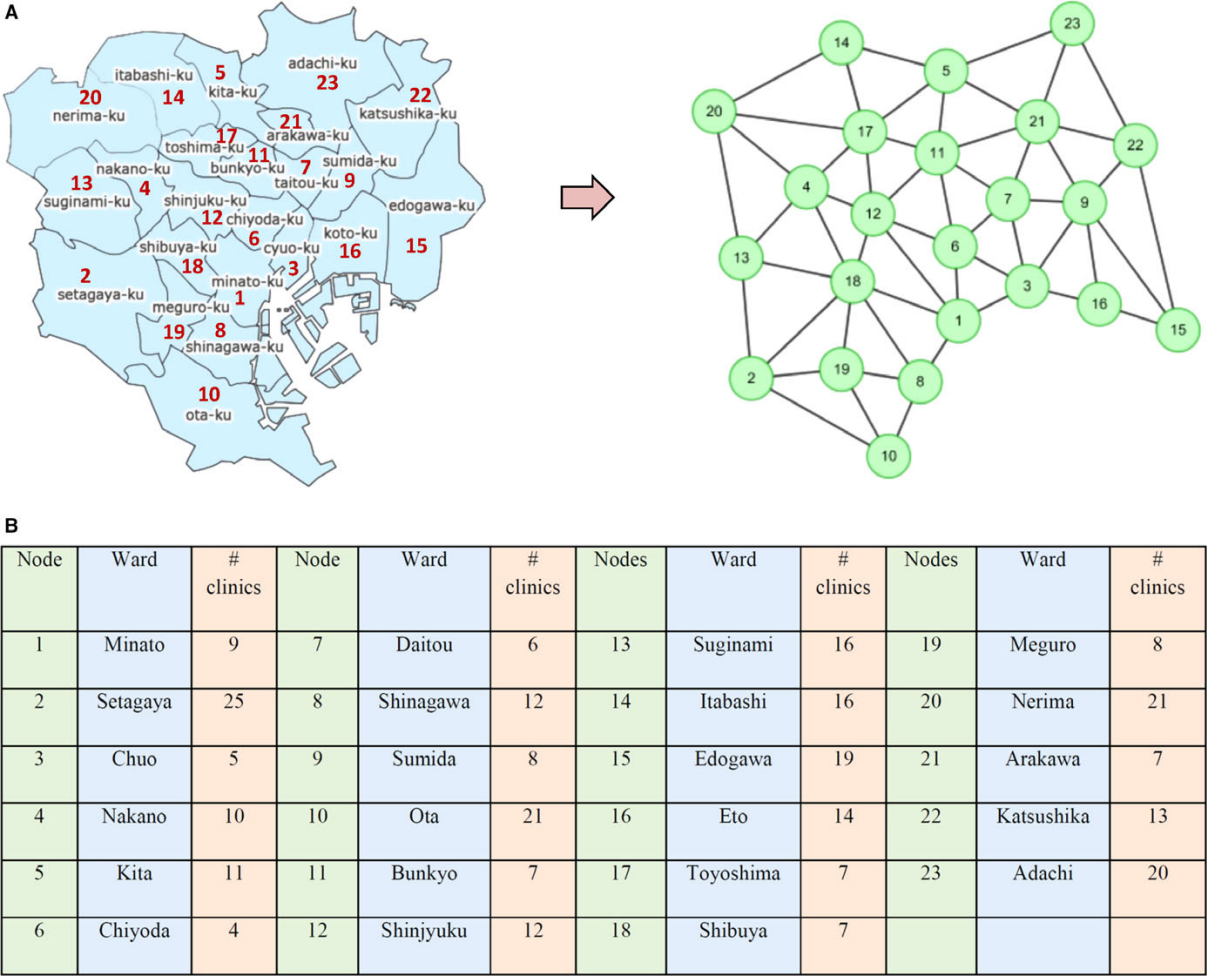
The network model for Tokyo city. A, Based on the geographic distribution of 23 wards and their adjacent relationship, a 23‐node neighboring network model is constructed. For each week, the average counts of clinic visiting within a ward were mapped to the corresponding node, through which we obtained a data matrix with 23 rows/wards and 376 columns/weeks. The detailed corresponding list between wards and nodes is shown in (B) (as of June 2018)

Given a network structure for the observed variables, an efficient method to detect DNM, called the landscape dynamic network marker (or landscape dynamic network biomarker), is proposed by employing the local‐landscape method on the basis of the three DNM statistic properties.^9^ Specifically, first we mapped the historic records of flu patients to the city network (Figure 2A). Second, the network was partitioned into many local networks. Each local network contained a centre node/ward and all of its first‐order neighbours based on the network structure. The local‐network index *I*‐score of a centre node at time point *t* for a local network with *n* members (ie, one centre node with *n*‐1 first‐order neighbouring nodes) was then calculated through the following definition:

$$I_{t}=|\Delta SD_{t}\left(in\right)|[|\Delta PCC_{t}\left(in\right)|+|\Delta PCC_{t}\left(out\right)\left|\right],$$

where

$$|\Delta SD_{t}\left(in\right)|=\frac{\sum_{i=1}^{n}|SD_{t}\left(i\right)-SD_{t-1}\left(i\right)|}{n}$$

is the average differential standard deviation (in absolute value) of the nodes inside the local network;

$$|\Delta PCC_{t}\left(in\right)|=\frac{\sum_{i=1,j=1}^{n}\left|PCC_{t}\left(i,j\right)-PCC_{t-1}\left(i,j\right)\right|}{n\times n}$$

is the average differential Pearson's correlation coefficient (in absolute value) inside the local network, i.e., both nodes *i* and *j* are in the same local network;

$$|\Delta PCC_{t}\left(out\right)|=\frac{\sum_{i=1,j=1}^{n}\left|PCC_{t}\left(i,j\right)-PCC_{t-1}\left(i,j\right)\right|}{n\times n}$$

is the average differential Pearson's correlation coefficient (in absolute value) between a member (node *i*) in the local network and that (node *j*) outside.

Theoretically, when the system approaches the tipping point, ie, *t* ∈*critical state, and t‐1* ∉ *critical state*, there are three cases for the local network of a centre node:

- In the local network, all the nodes (or nodes) are DNM members;
- In the local network, there are DNM and non‐DNM members;
- In the local network, all the nodes are non‐DNM members.

According to the three cases respectively, there are critical behaviours for a centre node shown as in Table 1.

**Table 1:** Local‐network index *I*‐score of a center node

Case	Members/nodes		*SD*_*t*_	|*ΔSD*_*t*_ (*in*)|	*PCC*_*t*_ (*in*)	|*ΔPCC*_*t*_ (*in*)|	*PCC*_*t*_ (*out*)	*|*∆*PCCout* (*t*)|	*I*_*t*_
1.	All DNM		↗	↗	↗	↗	D↗	↗	↗
							N↘		
2.	DNM and non‐DNM	D	↗	↗	D ↗	↗	D ↗	↗	↗
					N ↘	↗	N ↘	↗	
		N	→	0	D ↘	↗	D ↘	↗	
					N →	0	N →	0	
3.	All non‐DNM		→	0	→	0	D ↘	↗	0
							N→	0	

Notation: the system is near a tipping point, ie, it moves from time point *t*‐1 to *t,* with *t* ∈ *critical state, and t‐1* ∉ *critical state*.

1. “↗” represents the increase of the index; “↘” represents the decrease of the index; “→” represents that there is no significant change in the index.

2. “D” stands for the DNM members, or the PCC with DNM members; “N” stands for the non‐DNM nodes, or the PCC with non‐DNM members.

3. *SD*_*t*_ is the average standard deviation at time t; *PCC*_*t*_ (*in*) is the average Pearson's correlation coefficient between two nodes inside the local network; *PCC*_*t*_ (*out*) is the average Pearson's correlation coefficient between a node inside the local network and a node outside.

**Figure 3:**
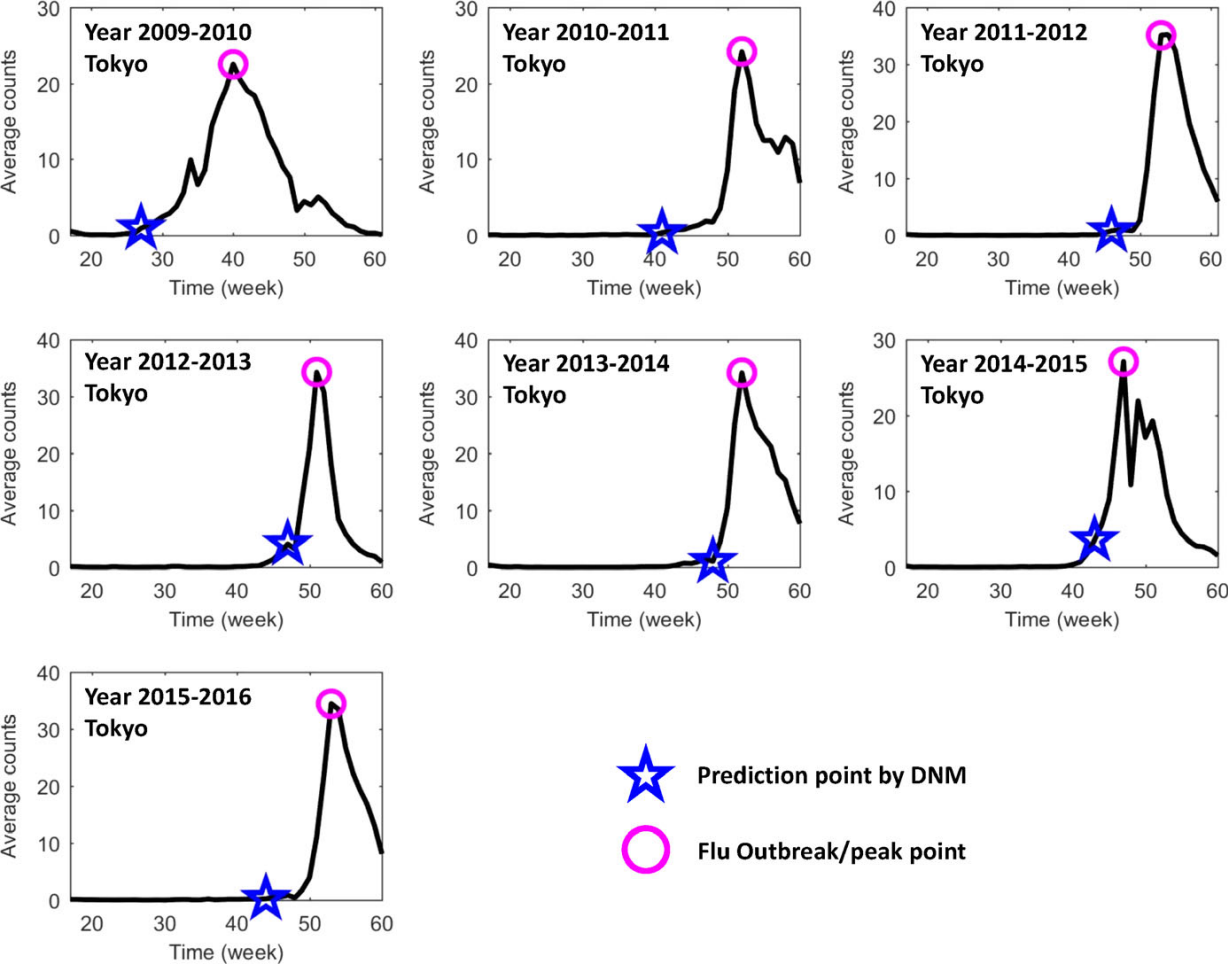
The prediction of annual seasonal influenza outbreak in Tokyo city between the years 2009 and 2016. Based on the public historical information of flu‐caused hospitalization between 1 March 2009 and 31 March 2016 in Tokyo, Japan, each seasonal influenza outbreak is predicted by DNM. The DNM score was calculated based on a 5‐wk sliding window scheme. In each figure, the y‐axis is the average number of patients in each clinic; the x‐axis represents a period spanning from the 17th week (the first week in May) to the 60th or 61st week (the last week in March). There are 52 weeks in years 2009 and 2011, and 53 wk in other years. The pink circle points to the peak of average patient counts, ie, the flu outbreak, and the blue star mark indicates the first warning time (tipping point) signalled by the DNM score. Clearly, DNM is able to predict the outbreak before the drastic increase of the patients

Thus, the network based index, *I*_*t*_, can quantitatively characterize the criticality of the state for each DNM member or node. Clearly, each node has an *I*_*t*_ value, and hence those *I*_*t*_ scores for all of nodes with the time evolution construct a landscape as shown in Figure 3. When the system approaches the critical state, *I*_*t*_ of each DNM node increases drastically based on the three statistic conditions of DNM, while *I*_*t*_ of other non‐DNM node may have no significant change. Obviously, during the critical transition, the DNM group has an ability to generate detectable early‐warning signals of the upcoming critical transition.

### 2.3 Data collecting and processing

#### 2.3.1 Data collection

The historical raw data of Tokyo region was downloaded through Tokyo Metropolitan Infectious Disease Surveillance Center (Link: http://survey.tokyo-eiken.go.jp/epidinfo/weeklyhc.do). The raw data of Hokkaido were downloaded through Hokkaido Infectious Disease Surveillance Center (Link: http://www.iph.pref.hokkaido.jp/kansen/501/data.html).

#### 2.3.2 Data normalization

For each ward or district, the raw data were averaged in terms of the total number of clinics within the ward/district. This normalization process is directly related to the population of each ward/district, since the population is roughly proportional to the number of clinics.

#### 2.3.3 Sliding window

The raw data were processed through window shift where window breadth is set as 5, that is, both the standard deviation and correlation coefficient are calculated based on the data within every 5 weeks.

## 3 RESULTS

### 3.1 Detecting the seasonal flu outbreak in Tokyo

The flu transmission dynamics before sudden outbreak is usually too complicated to be fully expressed mathematically in high‐dimensional spaces involving both biological systems and social systems. The drastic or a qualitative transition in a local system or network, from a normal state to an after‐outbreak state, corresponds to a so‐called bifurcation point in dynamical systems theory.^21^, ^23^ If the system is approaching a bifurcation point, it will eventually be constrained to a one‐ or two‐dimensional space (ie, the centre manifold in a generic sense), in which a dynamical system can be expressed in a very simple form. This is the theoretical basis for developing a general indicator that can detect the critical state of flu outbreak only based on the observed data.

As shown in Figure 1, we collected the historical longitudinal records of flu‐caused hospitalization from clinics distributed in 23 wards in Tokyo, Japan, between 1 January 2009 and 31 December 2016. The time evolution or dynamics of the hospitalization counts spanning from January 2009 to December 2015 in Tokyo is presented in Figure S1. During each year, the flu outbreak point is defined as the peak of the total hospitalization counts. To profile the flu transmission in the city, a 23‐node network is constructed according to the actual locations of 23 wards and their adjacent relationship (Figure 2).

Shown as in Figure 3, the early‐warning signals were detected by DNM method for each seasonal outbreak of influenza. It can be seen that the flu outbreaks around the early January is quite regular from years 2010 to 2015, while that in year 2009 is an exception. The novel H1N1 virus was detected in 2009 pandemic that was reported in Mexico first and then spreading around the world, which caused an early flu outbreak in Tokyo spanning from the 30th to 50th weeks (around October) of year 2009. Therefore, for each flu epidemic later developing into massive outbreak, the DNM score is sensitive and increases significantly at least 3 weeks (leading time) before the boost of hospitalization counts (Figure 3), indicating the emergence of the critical transition into a catastrophic pandemic.

**Figure 4:**
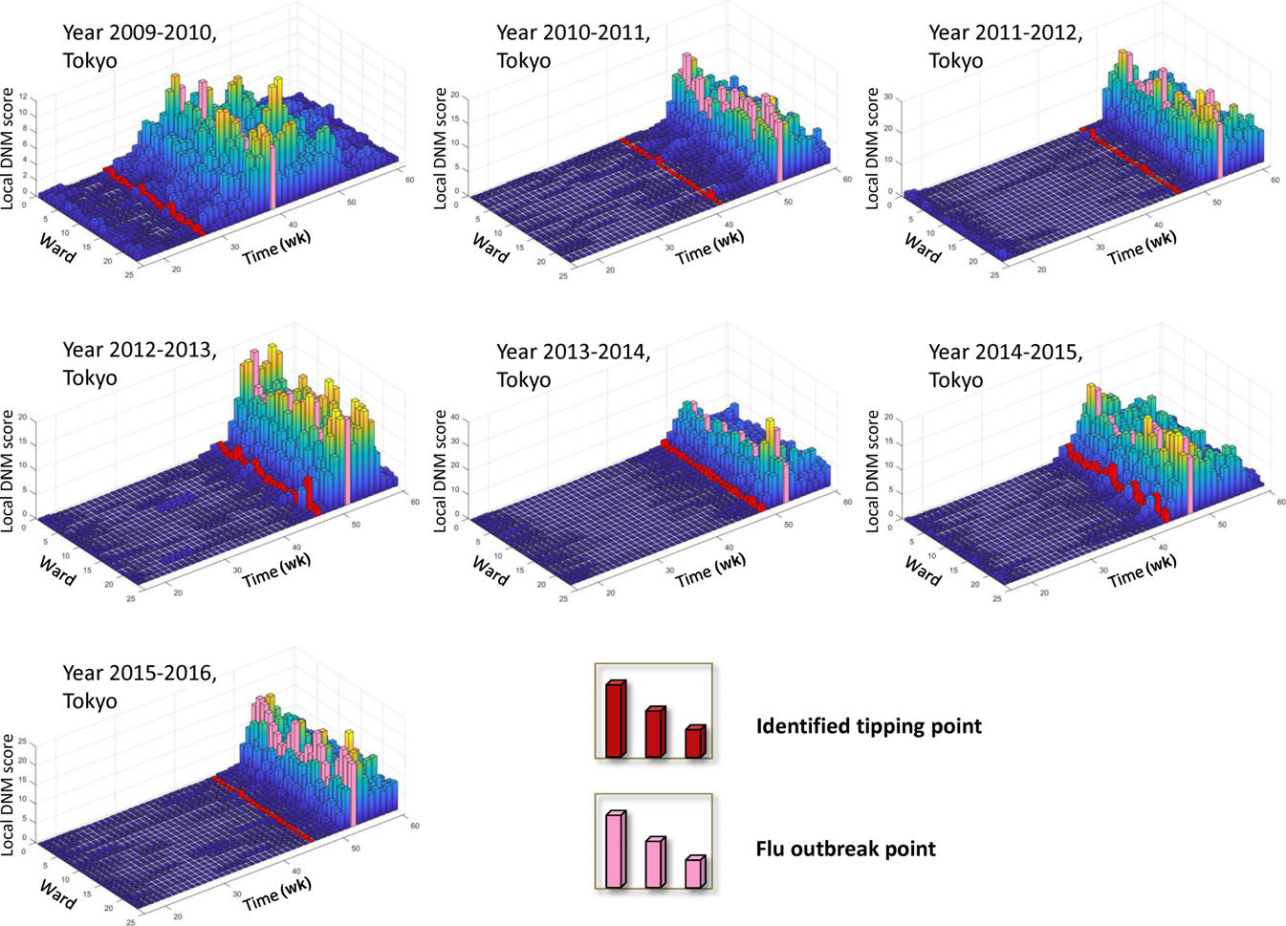
The local DNM scores or landscapes for 23 wards in Tokyo city between the years 2009 and 2015. In each landscape figure, the local DNM scores for 23 wards in Tokyo are presented annually. The red column points to the first emergence of the warning signal, while the pink column indicates the flu outbreak. The definition and calculation of local DNM score are shown in the Methods section. Clearly, the warning signals are earlier than the outbreaks

To better illustrate how DNM works, we show the local DNM landscape as in Figure 4. It can be seen that the first‐found DNM signals are about 3‐9 weeks ahead the flu outbreak point defined at the peak of hospitalization counts. The successful prediction of each flu outbreak suggests the robustness and effectiveness of DNM system in detecting the upcoming influenza outbreak based on the observed data.

**Figure 5:**
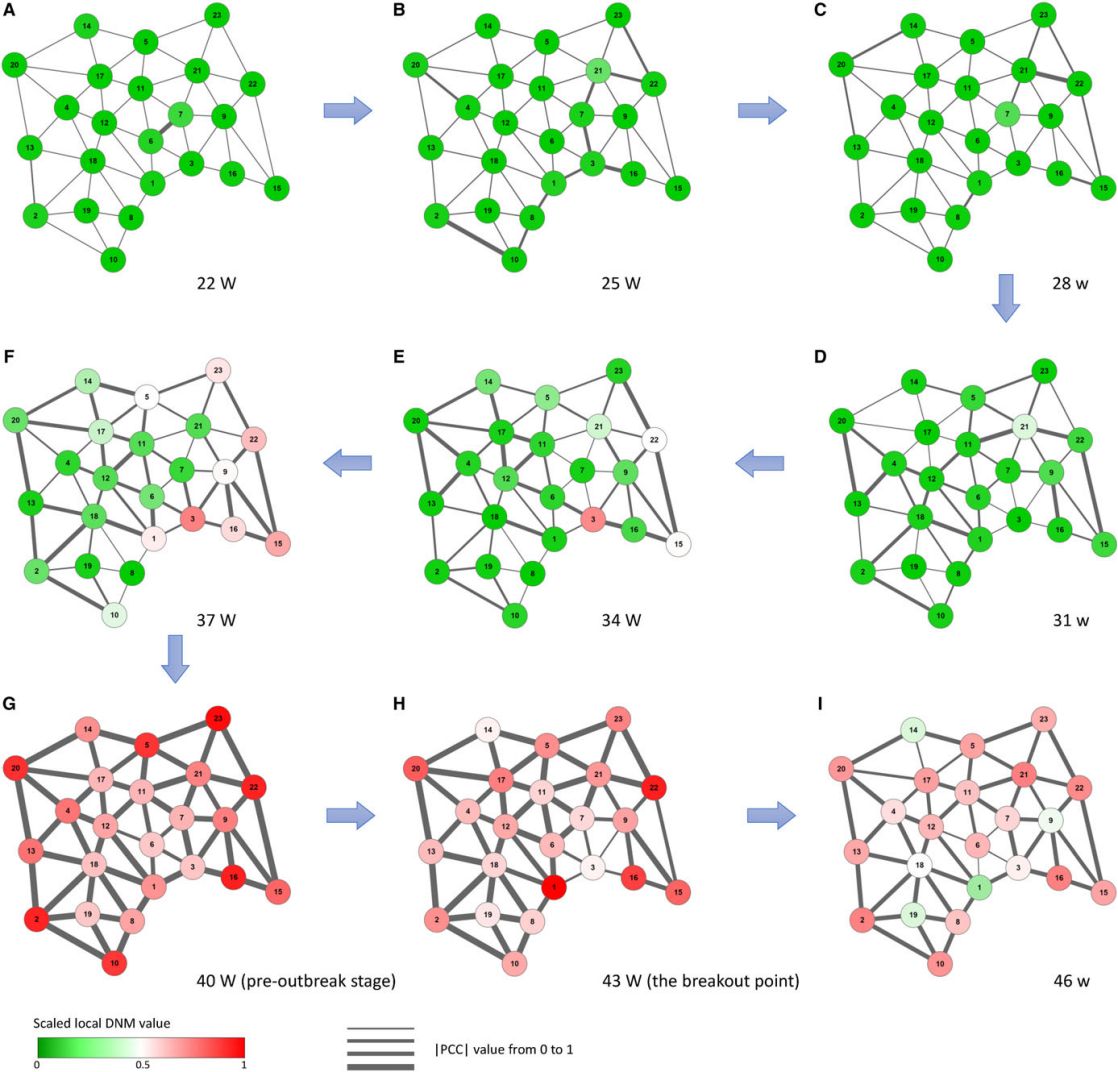
The dynamical evolution of flu‐progression network in Tokyo. Based on the local DNM scores for 23 wards in Tokyo during the year 2014, the dynamical evolution of flu‐progression network is presented, i.e., networks respectively in (A) the 22nd wk, (B) the 25th wk, (C) the 28th wk, (D) the 31st wk, (E) the 34th wk, (F) the 37th wk, (G) the 40th wk, (H) the 43rd wk, and (I) the 46th wk. The nodes are coloured by the scaled value of local DNM score, while the thickness of the edges represents the correlation between a pair of adjacent wards in Tokyo. It can be seen that there is no significant changes in the network (eg, the 22nd‐34th week) far from the flu outbreak caused by influenza A virus H3N2 in the 43rd week. However, when the system approaches the outbreak point (eg, the 40th week), there are tremendous changes in both nodes and edges, reflecting the obvious early‐warning signals of the upcoming outbreak, provided by the DNM system

Besides, we presented the dynamical evolution of local DNM scores in respect of ward‐network. Figure 5 shows the dynamics of the network of year 2014 in terms of local DNM scores. It can be seen that both the local DNM for each node and the correlation between each pair of adjacent wards are at a low‐level at the beginning of the process (the 22nd‐34th week). When the system approaches to the outbreak point, the signal arises to indicate the abnormal change in the whole network, that is, both the local DNM and the correlation between adjacent wards increases sharply, which are the necessary conditions of the DNM. Besides, it is seen from Figure 5E that the initial signal (the first red node) appears in Chuo Ward (node 3) in 34th week, which is consistent with the historical records (Table S1). The dynamical evolution of network shows that the DNM‐based system uncovers the epidemic situation and transmission trends, which better present the transmission dynamics at a system network level.

### 3.2 Application of DNM in Hokkaido region

As another DNM application to the influenza outbreak, we also applied the DNM to detect the early‐warning signals against flu outbreak in Hokkaido region, which is shown in Figures S2‐S5. It is seen from Figure S5 that there are 30 districts in Hokkaido region. From year 2009 to 2016, there are seven seasonal flu pandemics, among which the DNM‐based score provides early‐warning signals to six outbreaks (Figures S2 and S3). Figure S4 shows the dynamics of the region network of year 2014 in terms of local DNM scores.

### 3.3 Performance comparison with other methods

**Figure 6:**
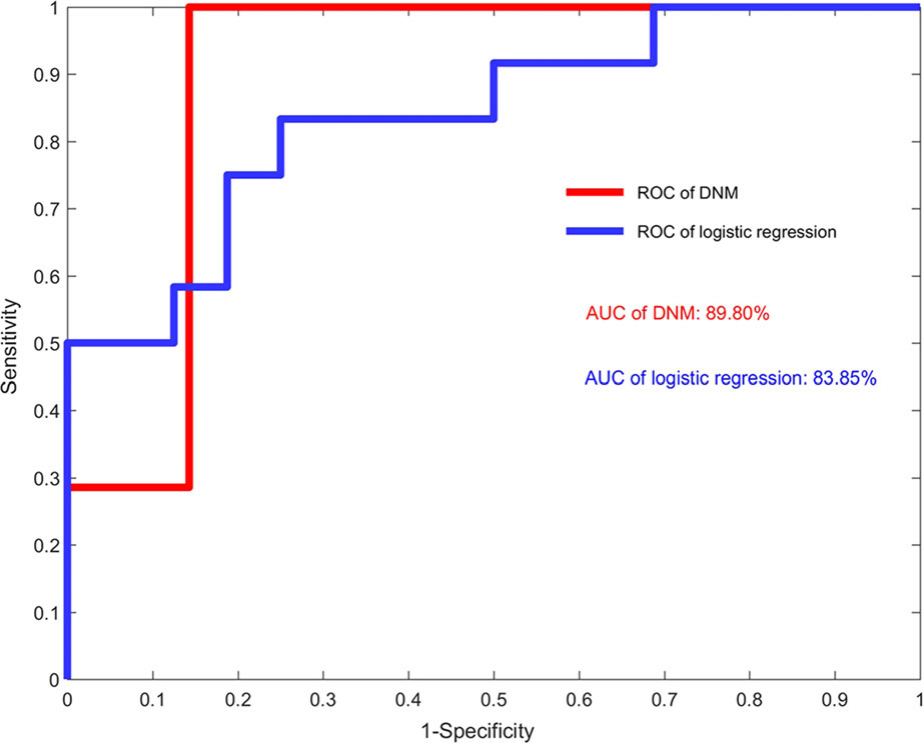
The performance of DNM‐based and machine‐learning‐based methods. It is seen that using only the hospitalization records, the DNM‐based surveillance system performs better than the logistic regression. The AUC of DNM is 0.898 while that of logistic regression is 0.839. The performance comparison is carried out based on the data of Tokyo. Note that the DNM‐based method generally has no overfitting problem due to the three statistic conditions of DNM without the training data, in contrast to the machine‐learning‐based methods that depend on the training data

The performances of DNM score is compared to other systems using machine learning algorithm (Figure 6). Specifically, a popular surveillance system of flu pandemic is based on logistic regression.^24^, ^25^, ^26^ It is clear from Figure 6 that given only hospitalization records, the DNM‐based system performs better than a system based on logistic regression.

Actually, the DNM method has natural advantage comparing with traditional machine learning algorithm in the following aspects. First, unlike machine learning based methods, DNM is a model free method, which solely depends on the three statistic conditions (see Methods section) and does not necessarily need procedures of training and testing. Second, as exhibited in the prediction of seasonal flu outbreaks, there is no feature selection in DNM strategy, thus avoid overfitting problem even if there are only small samples available.

## 4 DISCUSSION

The annual flu outbreak gives substantial impact to public health and the whole economy of Japan. Those outbreaks cause sickness, deaths, and quarantines, which are the three main factors which would result in reduction of human wellness as well as worker productivity. The deterioration in worker productivity would directly lead to a substantial impact in the economy. Additionally, transportation and production industries, for instance, would be heavily affected by the flu pandemics due to reasons such as border closures. According to ABARE, an Australian Research Agency, economy and industries in many countries will be seriously affected, for instance, Japan was predicted to have a decrease of 6.1% in gross domestic product (GDP) due to flu pandemic. From time to time, new strains emerge and cause global pandemics. To fight against influenza epidemic, it is of great importance to construct a monitoring system dependent solely on robust information, such as the hospitalization counts. In this study, DNM method shows its potential and power in detecting the early‐warning signals to the influenza outbreak, which may lead a new way of public real‐time surveillance for epidemic diseases.

Specifically, in line with our previous works on the tipping point or critical transition analysis of complex diseases with genomic datasets, we identified a pre‐outbreak state of influenza epidemic through mining the longitudinal clinic records. In particular, a major advantage of our method is that DNM can extract the dynamic information from high‐dimensional data, ie, simultaneously monitoring hundreds of districts and evaluating the outbreak risk by the landscape/local DNM score, as shown in Figures 4 and 5. It should be noted that the DNM‐warning system presented in this work is solely based on counts of hospitalization. Given more clinic information and new discriminant input patterns, DNM‐based monitoring system is expected to reliably predict the flu outbreak in terms of sensitivity and accuracy. The program of this work is available on the request, and can also be obtained from http://sysbio.sibcb.ac.cn/flu-outbreak-prediction, a website that incorporates data gathering and processing.

## CONFLICT OF INTEREST

The authors declare that they have no competing interests.

## AUTHOR CONTRIBUTIONS

RL and EC conceived the project; RL, LC, and XZ supervised the project. EC, PC, and RL performed computational and analysis. All authors wrote the manuscript. All authors read and approved the final manuscript.

## References

[1] Charu V , Viboud C , Simonsen L , et al. Influenza‐related mortality trends in Japanese and American seniors: evidence for the indirect mortality benefits of vaccinating schoolchildren. PLoS ONE. 2011;6(11):e26282. 22087226 10.1371/journal.pone.0026282 PMC3210121

[2] Fleming DM , Cross KW , Pannell RS . Influenza and its relationship to circulatory disorders. Epidemiol Infect. 2005;133(2):255‐262. 15816150 10.1017/s0950268804003231 PMC2870244

[3] Thompson MG , Shay DK , Zhou H , et al. Estimates of deaths associated with seasonal influenza‐United States, 1976‐2007. Morb Mortal Wkly Rep. 2010;59(33):1057‐1062. 20798667

[4] Molinari NAM , Ortega‐Sanchez IR , Messonnier ML , et al. The annual impact of seasonal influenza in the US: measuring disease burden and costs. Vaccine. 2007;25(27):5086‐5096. 17544181 10.1016/j.vaccine.2007.03.046

[5] Kelso JK , Milne GJ , Kelly H . Simulation suggests that rapid activation of social distancing can arrest epidemic development due to a novel strain of influenza. BMC Public Health. 2009;9(1):117. 19400970 10.1186/1471-2458-9-117 PMC2680828

[6] Zhong NS , Zeng GQ . Our strategies for fighting severe acute respiratory syndrome (SARS). Am J Respir Crit Care Med. 2003;168(1):7‐9. 12773318 10.1164/rccm.200305-707OE

[7] Wilson K , Brownstein JS . Early detection of disease outbreaks using the Internet. Can Med Assoc J. 2009;180(8):829‐831. 19364791 10.1503/cmaj.090215 PMC2665960

[8] Woodall J . Official versus unofficial outbreak reporting through the Internet. Int J Med Informatics. 1997;47:31‐34. 10.1016/s1386-5056(97)00079-8 9506388

[9] Chen L , Liu R , Liu ZP , Li MY , Aihara K . Detecting early‐warning signals for sudden deterioration of complex diseases by dynamical network biomarkers. Sci Rep. 2012;2:342. 22461973 10.1038/srep00342 PMC3314989

[10] Liu R , Wang X , Aihara K , Chen L . Early diagnosis of complex diseases by molecular biomarkers, network biomarkers, and dynamical network biomarkers. Med Res Rev. 2014;34:455‐478. 23775602 10.1002/med.21293

[11] Richard A , Boullu L , Herbach U , et al. Single‐cell‐based analysis highlights a surge in cell‐to‐cell molecular variability preceding irreversible commitment in a differentiation process. PLoS Biol. 2016;14:e1002585. 28027290 10.1371/journal.pbio.1002585 PMC5191835

[12] Mojtahedi M , Skupin A , Zhou J , et al. Cell fate decision as high‐dimensional critical state transition. PLoS Biol. 2016;14:e2000640. 28027308 10.1371/journal.pbio.2000640 PMC5189937

[13] Lesterhuis WJ , Bosco A , Millward MJ , et al. Dynamic versus static biomarkers in cancer immune checkpoint blockade: unravelling complexity. Nat Rev Drug Discov. 2017;16:264‐272. 28057932 10.1038/nrd.2016.233

[14] Li M , Zeng T , Liu R , Chen L . Detecting tissue‐specific early warning signals for complex diseases based on dynamical network biomarkers: study of type 2 diabetes by cross‐tissue analysis. Brief Bioinform. 2014;15:229‐243. 10.1093/bib/bbt027 23620135

[15] Liu R , Yu X , Liu X , et al. Identifying critical transitions of complex diseases based on a single sample. Bioinformatics. 2014;30:1579‐1586. 24519381 10.1093/bioinformatics/btu084

[16] Liu R , Li M , Liu ZP , et al. Identifying critical transitions and their leading biomolecular networks in complex diseases. Sci Rep. 2012;2:813. 23230504 10.1038/srep00813 PMC3517980

[17] Yang B , Li M , Tang W , et al. Dynamic network biomarker indicates pulmonary metastasis at the tipping point of hepatocellular carcinoma. Nat Commun. 2018;9(1):678. 29445139 10.1038/s41467-018-03024-2 PMC5813207

[18] Liu X , Liu R , Zhao XM , Chen L . Detecting early‐warning signals of type 1 diabetes and its leading biomolecular networks by dynamical network biomarkers. BMC Med Genomics. 2013;6(suppl 2):S8. 10.1186/1755-8794-6-S2-S8 PMC3654886 23819540

[19] Chen P , Liu R , Li Y , Chen L . Detecting critical state before phase transition of complex biological systems by hidden Markov model. Bioinformatics. 2016;32:2143‐2150. 27153710 10.1093/bioinformatics/btw154

[20] Chen P , Li Y , Liu X , Liu R , Chen L . Detecting the tipping points in a three‐state model of complex diseases by temporal differential networks. J Transl Med. 2017;15(1):217. 29073904 10.1186/s12967-017-1320-7 PMC5658963

[21] Chen P , Liu R , Aihara K , Chen L . Identifying critical differentiation state of MCF‐7 cells for breast cancer by dynamical network biomarkers. Front Genet. 2015;6:252. 26284108 10.3389/fgene.2015.00252 PMC4516973

[22] Murray JD . Mathematical Biology. New York, NY: Springer; 1993.

[23] Gilmore R . Catastrophe Theory for Scientists and Engineers. New York, NY: Dover; 1981.

[24] Pfeiffer DU , Minh PQ , Martin V , et al. An analysis of the spatial and temporal patterns of highly pathogenic avian influenza occurrence in Vietnam using national surveillance data. Vet J. 2007;174(2):302‐309. 17604193 10.1016/j.tvjl.2007.05.010

[25] Boivin G , Hardy I , Tellier G , et al. Predicting influenza infections during epidemics with use of a clinical case definition. Clin Infect Dis. 2000;31(5):1166‐1169. 11073747 10.1086/317425

[26] Fang LQ , de Vlas SJ , Liang S , et al. Environmental factors contributing to the spread of H5N1 avian influenza in mainland China. PLoS ONE. 2008;3(5):e2268. 18509468 10.1371/journal.pone.0002268 PMC2386237

