# Supplementary material for: Detecting early‐warning signals of influenza outbreak based on dynamic network marker
Source: J Cell Mol Med. 2018 Oct 19;23(1):395–404. doi: 10.1111/jcmm.13943 (PMC6307766; doi:10.1111/jcmm.13943)
Supplement: Supplementary file 1 [file JCMM-23-395-s001.docx]

**Supplementary Information of Detecting early-warning signals of influenza outbreak based on dynamic network marker**

Pei Chen, Ely Chen, Luonan Chen, Xianghong Jasmine Zhou, Rui Liu

**Supplementary Figures**


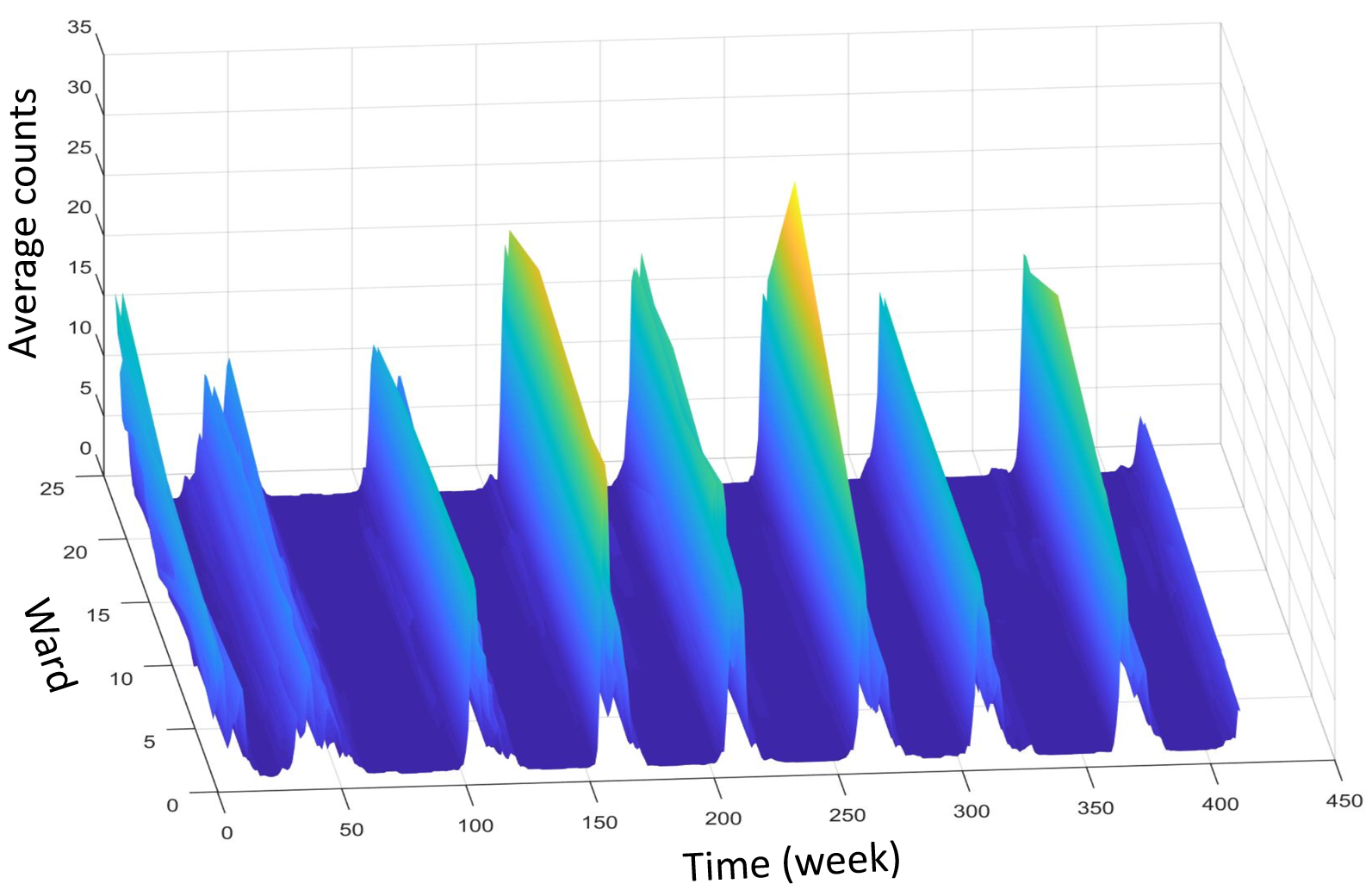


**Figure S1: The time evolution or dynamics of the hospitalization counts spanning from January 2009 to December 2016 in Tokyo.**


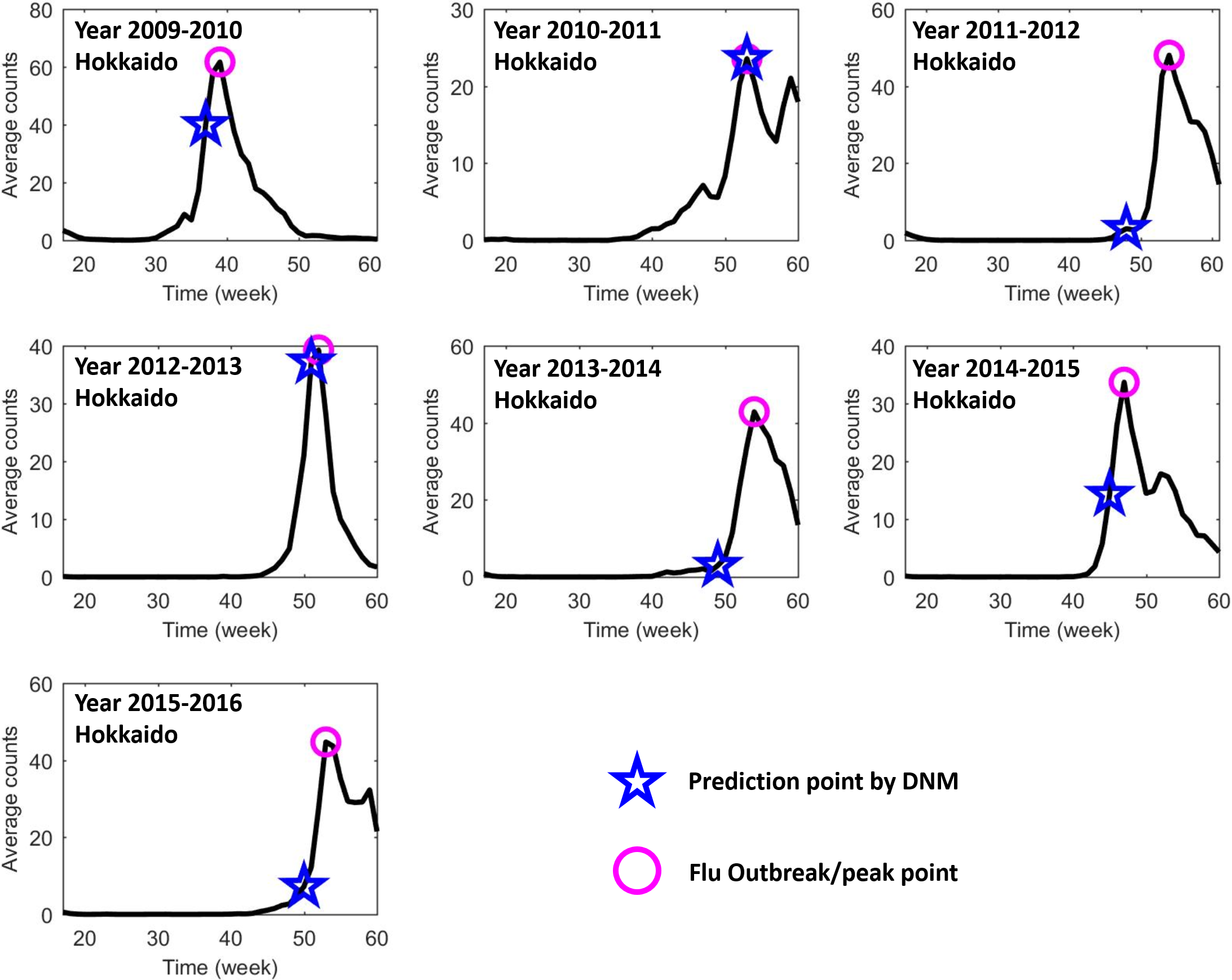


**Figure S2: The detection of annual seasonal influenza outbreak in Hokkaido region between year 2009 and 2016.** Based on the public historical information3 of flu-caused hospitalization between January 1, 2009 and March 31, 2016 in Hokkaido, Japan, each seasonal influenza outbreak is predicted by DNM. The DNM score was calculated based on a 5-week sliding window scheme. In each figure, the y-axis is the average number of patients in each clinic, the x-axis represents a period spanning from the 17^th^ week (the first week in May) to the 60^th^ or 61^th^ week (the last week in March). The red circle points the peak of average patient counts, i.e., the flu outbreak, and the blue star mark indicates the first warning time (tipping point) signaled by DNM score.


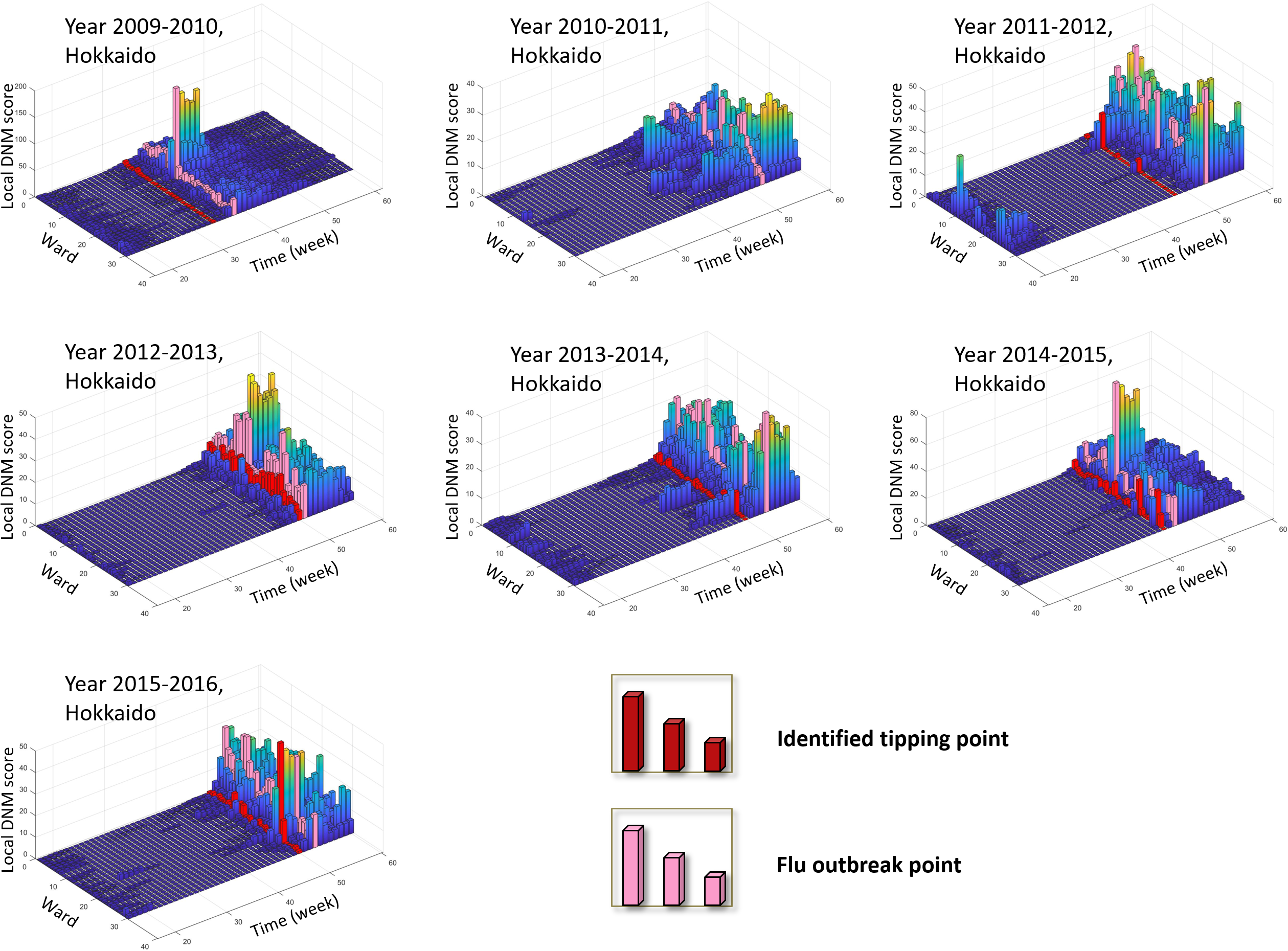


**Figure S3: The local DNM scores for 30 districts in Hokkaido region between year 2009 and 2015.** In each landscape figure, the local DNM scores respectively for 30 districts in Hokkaido are presented. The red column points the first emergence of warning signal, while the pink column indicates the flu outbreak.


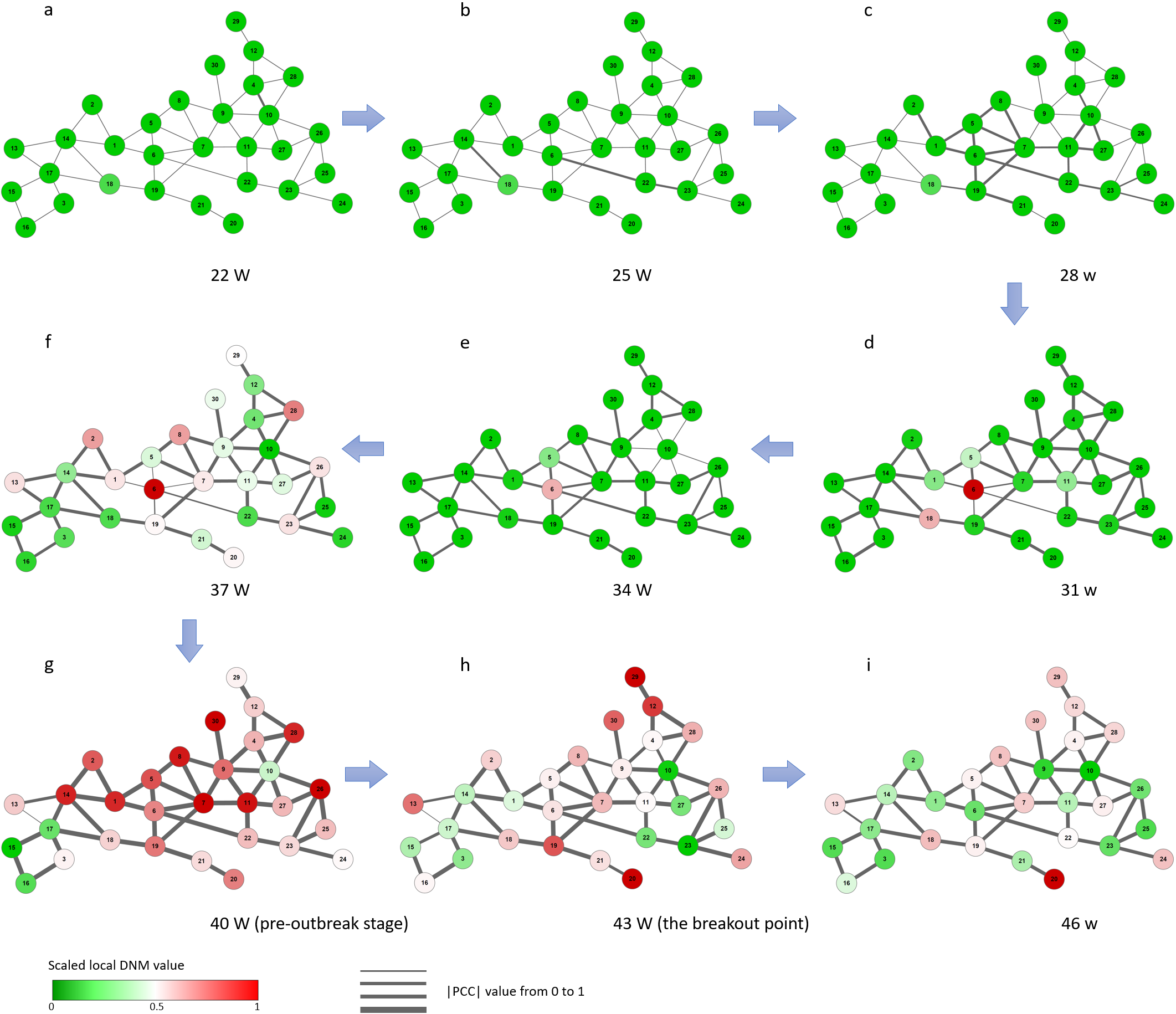


**Figure S4: The time evolution of flu-progression network in Hokkaido.** Based on the local DNM scores for 30 districts in Hokkaido during year 2014, the dynamical evolution of flu-progression network is presented, i.e., networks respectively in (a) the 22nd week, (b) the 25th week, (c) the 28th week, (d) the 31st week, (e) the 34th week, (f) the 37th week, (g) the 40th week, (h) the 43rd week, and (i) the 46th week. The nodes are colored by the scaled value of local DNM score, while the thickness of edges represents the correlation between a pair of adjacent wards in Tokyo. It can be seen that there is no significant change in the network far from the flu outbreak caused by influenza A virus H3N2 in the 43th week. However, when the system approaches the outbreak point, there are tremendous changes in both nodes and edges, reflecting the obvious early-warning signals provided by DNM system.


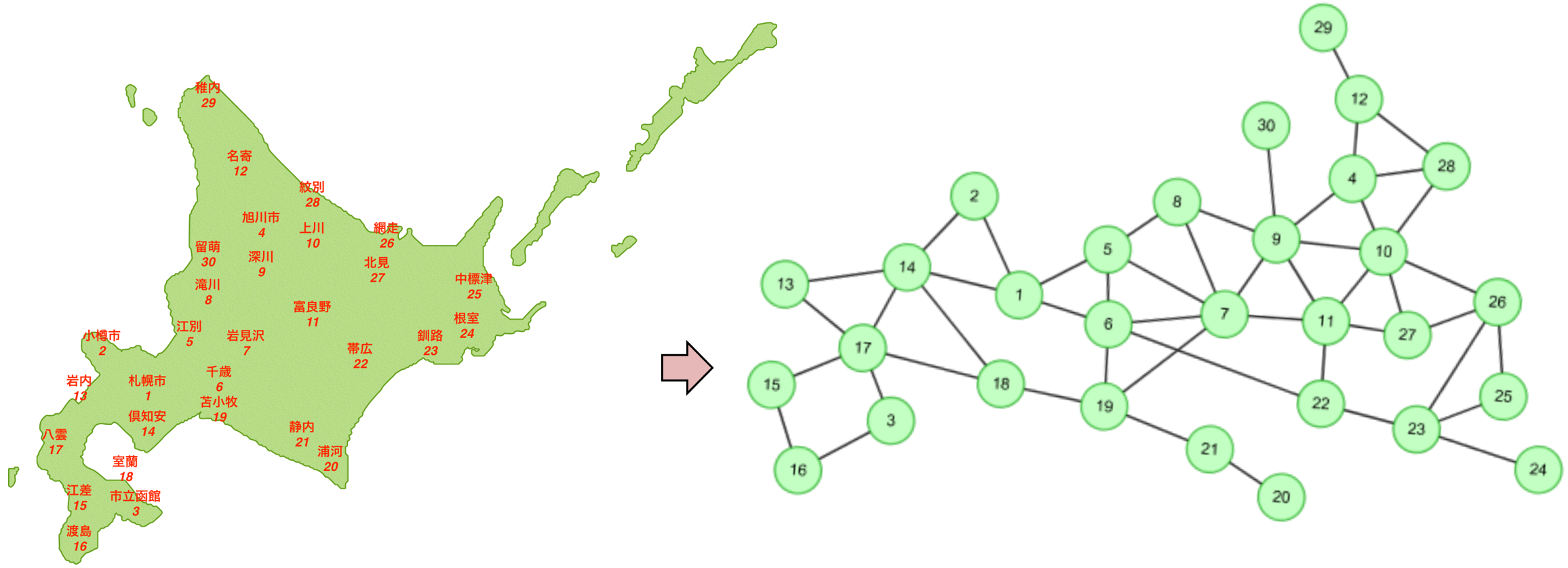


**Figure S5: The network for Hokkaido region.** Based on the geographic distribution of 30 districts and their adjacent relationship, a 30-node network model is constructed for analysis.

**Supplementary Table S1. The major outbreak virus type in Tokyo city**

| Year | Major outbreak virus type | Initial found ward |
| --- | --- | --- |
| 2009-2010 | A(H1N1)pdm09 | Ota Ward |
| 2010-2011 | A(H1N1)pdm09 | Sumida Ward |
| 2011-2012 | A(H3N2) | Arakawa Ward |
| 2012-2013 | A(H3N2) | Sumida Ward |
| 2013-2014 | A(H1N1)pdm09 | Chuo Ward |
| 2014-2015 | A(H3N2) | Edogawa Ward |
| 2015-2016 | A(H1N1)pdm09 | Daito Ward |
